# An augmented reality system for image guidance of transcatheter procedures for structural heart disease

**DOI:** 10.1371/journal.pone.0219174

**Published:** 2019-07-01

**Authors:** Jun Liu, Subhi J. Al’Aref, Gurpreet Singh, Alexandre Caprio, Amir Ali Amiri Moghadam, Sun-Joo Jang, S. Chiu Wong, James K. Min, Simon Dunham, Bobak Mosadegh

**Affiliations:** Dalio Institute of Cardiovascular Imaging, New York Presbyterian Hospital and Weill Cornell Medicine, New York, United States of America; Beijing University of Technology, CHINA

## Abstract

The primary mode of visualization during transcatheter procedures for structrural heart disease is fluoroscopy, which suffers from low contrast and lacks any depth perception, thus limiting the ability of an interventionalist to position a catheter accurately. This paper describes a new image guidance system by utilizing augmented reality to provide a 3D visual environment and quantitative feedback of the catheter’s position within the heart of the patient. The real-time 3D position of the catheter is acquired via two fluoroscopic images taken at different angles, and a patient-specific 3D heart rendering is produced pre-operatively from a CT scan. The spine acts as a fiduciary land marker, allowing the position and orientation of the catheter within the heart to be fully registered. The automated registration method is based on Fourier transformation, and has a high success rate (100%), low registration error (0.42 mm), and clinically acceptable computational cost (1.22 second). The 3D renderings are displayed and updated on the augmented reality device (i.e., Microsoft HoloLens), which can provide pre-set views of various angles of the heart using voice-command. This new image-guidance system with augmented reality provides a better visualization to interventionalists and potentially assists them in understanding of complicated cases. Furthermore, this system coupled with the developed 3D printed models can serve as a training tool for the next generation of cardiac interventionalists.

## Introduction

**Fig 1:**
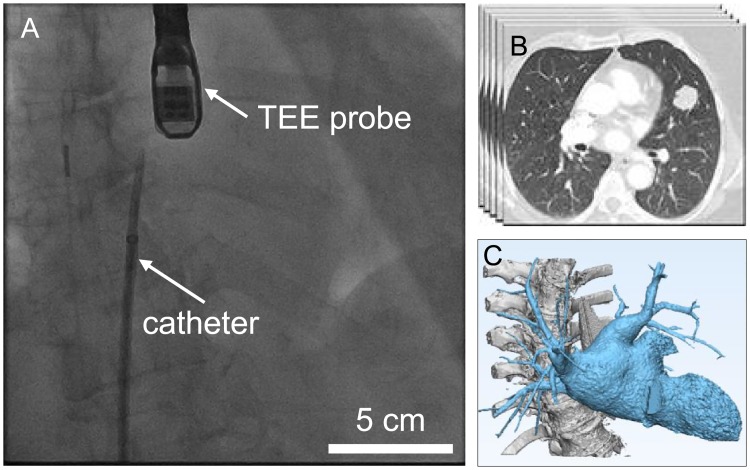
Imaging modalities in cardiovascular interventions. A: The heart is transparent in real-time X-ray fluoroscopic images. B: Pre-operative CT scan images provide a high contrast for understanding heart anatomy with (C) the 3D reconstruction.

Transcatheter procedures are the predominant and increasingly favored treatment approach in a wide variety of structural heart disease, including atrial septal defect and patent foramen ovale closure, valvular repair/replacement, left atrial appendage closure and deployment of hemodynamic monitoring devices [1]. These procedures are typically performed under X-ray fluoroscopy since it is the most common imaging modality, provides real-time imaging, and can readily visualize radiopaque markers on transcatheter devices to help locate equipment position [2]. However, while one can identify the silhouette of the heart using fluoroscopy, the intracardiac structures are transparent, and therefore contrast agents, which transiently opacify the structure of interest, are used to visualize the relative position of the catheter to surrounding tissues. Furthermore, fluoroscopy only provides a 2D projection of the heart, catheter and other devices, and therefore no information of depth is provided (Fig 1A).

Besides fluoroscopy imaging, echocardiography can directly image heart tissue and blood flow, and is often used as a complementary imaging modality [3]. Although recent literature reports that transesophageal echocardiography (TEE) has been used to provide intra-operative 2D and 3D visual feedback, this technology requires skilled operators, and uses TEE equipment not readily available in all hospitals [4]. The limitations of these imaging techniques increase the complexity and uncertainty of current procedures, requiring the interventionalist to estimate the 3D position and orientation of the catheter/device by analyzing images from multiple imaging angles and modalities.

Preoperative imaging modalities, such as computed tomography (CT) and magnetic resonance (MR) imaging, which provide detailed anatomical information in 3D (Fig 1B & 1C), can be displayed on separate screens or overlaid on real-time imaging modalities (i.e., fluoroscopy and echocardiography) to improve image-guided interventions. Moreover, the preoperative images can be processed by applying novel deep learning methods (e.g., CSRNet) to provide the quantification results for the segmentation of cardiovascular structures [5]. However, the method of displaying hybrid images provides little additional information and often obstructs the view of the real-time image during the procedure [6]. Furthermore, all of these images are displayed on 2D screens, which fundamentally mitigate any depth perception, thus limiting the ability to perceive the position and angle of a catheter within the 3D heart rendering.

Automated image registration of different modalities is a non-trivial endeavor but useful for a variety of applications, ranging from diagnostics and surgical planning, to image-guided surgery and post-procedural evaluation of therapeutic outcomes [7]. Literature survey on medical image registration indicates that a variety of algorithms have been developed, including intensity- [8], gradient- [9] and feature-based methods [10]. The intensity-based image registration has been applied for registering CT and MR images by maximizing the mutual information between two images [11]. However, pixel intensities may vary dramatically due to the inconsistent imaging parameters in different modalities. Compared to intensity, image gradients are more stable factors because the contrast between the target anatomy and surrounding regions (e.g., bone vs. soft tissues) remains similar across different imaging modalities. Therefore, other researchers proposed a novel method based on the differential total variation for registration of MR images [12]. Beyond gradient-based registration, feature descriptors (e.g., SIFT, SURF, and BRIEF) were also used for registration of medical images [13, 14]. These feature-based registration methods are preferred to intensity- or gradient-based registration methods, due to its broader applicability, but the challenge of accurately segmenting cardiac features from different image modalities has yet to be addressed. For example, the heart is visible in CT scans while it appears transparent or with low-contrast in fluoroscopic images due to the restricted X-ray energy used to ensure the safety of the patients and interventionalists.

Augmented reality, as an emerging technology for providing enhanced visualization, has attracted increasing interest in the medical community. For example, OpenSight is the first FDA cleared AR system to be used for pre-operative planning of surgeries. The pioneering AR systems for medical use are mainly focused on improving the visualization in orthopedic surgeries [15, 16] and neurosurgeries [17, 18]. These systems, however, cannot be used for cardiac interventions because they do not provide real-time monitoring or quantitative feedback, which is critical to instruct and/or aid physicians for positioning catheters and devices [19].

**Fig 2:**
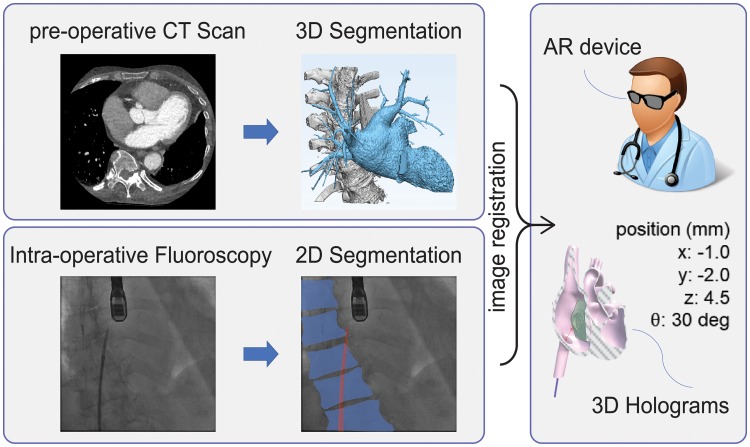
Overall procedure of the proposed AR assisted guidance system.

To address these limitations, we propose a novel approach for image guidance which displays, in real-time, high-resolution 3D holographic renderings of the catheter and patient’s heart using augmented reality devices. The overall procedure of the proposed AR assisted guidance system is shown in Fig 2. The geometry of the patient’s heart is generated (prior to the procedure) through the segmentation of cardiac CT scans. The real-time position and orientation of the catheter are automatically detected by processing fluoroscopic images that are captured from two angles. By registering the fluoroscopic images with the CT scans, the heart and catheter renderings are set into the same coordinate system and displayed on the AR device. The registration of the fluoroscopic images and CT scans is achieved by using the spine as a universal fiduciary marker because the spine is stationary and relatively clear in both imaging modalities.

This paper specifically reports the demonstration of the proposed AR image- guidance system on a 3D printed model of a heart and spine as segmented from a human CT scan. We demonstrate optimized methods for segmentation of the spine from fluoroscopic images, and registration of the two image modalities to provide a single coordinate system for rendering the catheter’s position in AR. Based on registration results, the detected catheter is rendered inside the heart model as 3D holograms using the AR devices to provide quantitative visual feedback for guiding the interventional procedure. Although the printed heart model is static, it serves as a proof of principle that the AR-guidance system may be used for both procedural and training purposes. Future work will focus on developing dynamic heart models that better recapitulate cardiac motion from both respiration and contraction.

## Materials and methods

The use of the medical images in this study was approved by the institutional review board (IRB) at Weill Cornell Medicine. The informed consent requirement was waived as the patients’ information were de-identified prior to the study.

### Segmentation of 3D anatomical structure from CT images

**Fig 3:**
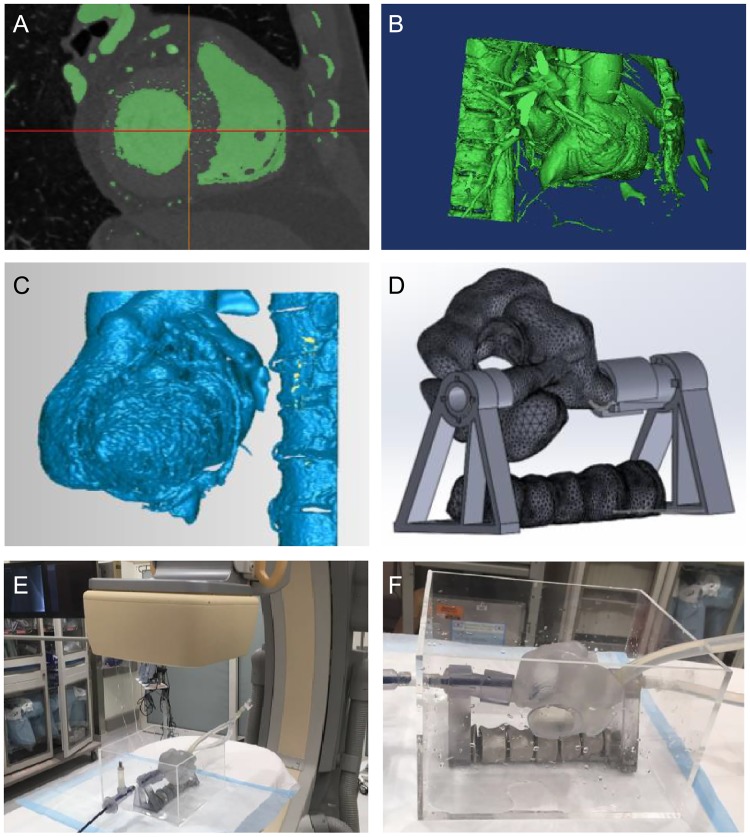
Segmentation of anatomical structures. A: A slice of CT scans shows the segmented region after thresholding. B: 3D reconstruction of the heart and spine by selecting the largest object and filling inner holes. C: The 3D models are cleaned by deleting peripheral blood vessels and bones. D: 3D models are further modified and fabricated using a 3D printer. E&F: The 3D printed model is tested under the X-ray fluoroscopy machine.

The 3D anatomical structures were segmented from diagnostic CT scans to provide a direct visualization of the patient’s heart during the interventional procedure. The original CT images were first imported as a Dicom file format into a 3D medical image processing software (i.e., Materialise Mimics). Each slice of the CT images was then binarized by manually selecting an appropriate threshold value according to the target anatomical structure of interest (Fig 3A). The threshold processing typically generates a rough segmentation that contains many isolated 3D objects due to the low contrast and low resolution of the CT images (Fig 3B). To remove the noise, only the largest object is selected, and its inner holes are filled to generate the 3D model. The largest object was then modified by setting a 3D mask and further adding/removing target regions for each slice. The reconstructed 3D model was finally smoothed and exported as an STL file. Both the segmented heart volume and spine were combined in the 3-matics software to unify all meshes in the same coordinate system.

After the segmentation from the volumetric CT images, the 3D CADs (computer aided designs) were imported into another editing software (i.e., Geomagic Wrap, 3D Systems Corporation) to further trim away the undesired structures including ribs, chest bones, or small peripheral blood vessels (Fig 3C & 3D). These features are trimmed to produce models that are practical to 3D print and visualize in the AR environment. For specific percutaneous transcatheter procedures (e.g., transseptal puncture), a preplanned path and target rings were added to the CADs in order to provide visual guidance to the interventionalists.

### Spine detection in fluoroscopic and CT images

In order to detect the catheter’s position and provide the 3D visual guidance in real time, the first step is to register the coordinate systems of the fluoroscopic images and CT scan. The spine was selected as a universal fiduciary marker because it is relatively clear in both imaging modalities with minimal or no movement and is stable between the time a preoperative CT is acquired and a transcatheter procedure is performed. Since the variations in positioning of the patients can affect the geometry and location of the spine, the patients are required to lie in supine position for both CT imaging and X-ray fluoroscopic procedures to ensure the consistency of spine appearance. Furthermore, our algorithm does not make any assumption regarding the orientation of the spine, and therefore it will minimize the sensitivity to changes due to patient positioning. However, this effect needs to be further studied in future work where images of a single patient with minor variation in positioning can be obtained.

In the experiments, five thoracic vertebrae (T4-T8) are selected as the fiduciary marker because they are close to the atriums of the heart and are visible during the transseptal puncture procedure. In addition, the vertebral bodies are selected as the intrinsic fiducial marker because they are the most visible in a fluoroscopic image, and thus minimizes the needed amount of radiation.

**Fig 4:**
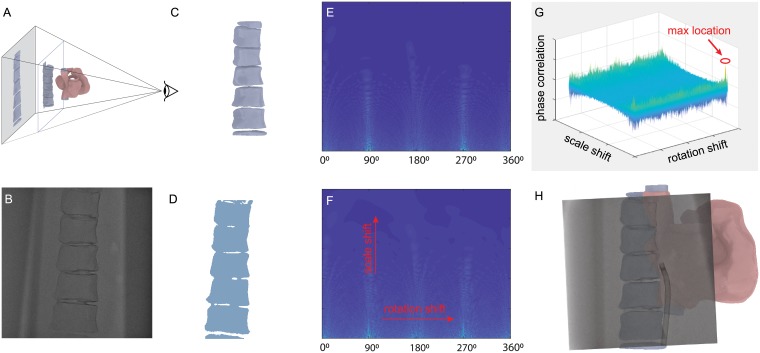
The Fourier based registration method. A: A projectional view of the 3D model that is reconstructed from CT images. B: The original fluoroscopic image is taken at RAO30. C: Only the spine image is created from the 3D model. D: The spine image detected from the fluoroscopic image. E-F: The polar-logarithmic transformed Fourier images corresponding to C-D; the rotation and scale factors are converted to the translations in X and Y axis. G: The phase correlation plot shows the maximum point is located at the position corresponding to the rotation and scale shifts. H: The overlaid image shows the final registration result.

Spine detection in CT images was achieved by taking a projection view from the 3D segmented models, such that a 2D image of the spine with a similar outline as the fluoroscopic image was obtained (Fig 4A). The projection angle was set equivalent to the capturing angle of the c-arm as recorded in the meta-data of the fluoroscopic image. Fig 4C shows a projection view of the spine created at Right Anterior Oblique view 30 degree (i.e., RAO30). However, since the patient may not be perfectly flat on the table, the fluoroscopic image and projection image of the spines are registered using a Fourier-based method.

To detect the spine from fluoroscopy, the noise of the original image (Fig 4B) was first reduced through the Gaussian smoothing method and binarized by applying Otsu’s adaptive thresholding. A bounding box calculated from the largest object was used to generate a region of interest for the refined binarization. A morphological close operation is then performed to remove noise and small particles that may be present in the region of interest. The segmented spine from a fluoroscopic image is shown in Fig 4D.

### Image registration based on fourier transform

Since the spine is a rigid object and its relative position inside the patient is consistent, a rigid registration is used to determine the transformation matrix between the fluoroscopic images and the 3D heart model. A 2D projection image of the segmented 3D spine model is denoted as the fixed reference image (*I*_*c*_), whereas the X-ray fluoroscopic images captured at the same angle is denoted as the moving image (*I*_*f*_). The registration of the two image modalities enables to determine the transform matrix (*T*_*f*_) such that the catheter and heart can be put into a single coordinate system with the scaling factor *k*, rotation angle *θ*, and translation (*t*_*x*_, *t*_*y*_),

$$P_{c}=[\begin{array}{c} x_{c}\\ y_{c}\\ 1 \end{array}]=T_{f}P_{f}=[\begin{array}{ccc} k\;\text{cos}\;\alpha & k\;\text{sin}\;\alpha & t_{x}\\ -k\;\text{sin}\;\alpha & k\;\text{cos}\;\alpha & t_{y}\\ 0 & 0 & 1 \end{array}][\begin{array}{c} x_{f}\\ y_{f}\\ 1 \end{array}]$$ (1)

where, *P*_*c*_ and *P*_*f*_ are the spine positions in CT and fluoroscopic images, respectively. In order to decouple the scale, rotation and translation factors, the spine images are first processed through 2D Fourier transformation. According to the properties of the Fourier transform, the transformed images ***F***_*c*_ and ***F***_*f*_ are related by

$$F_{c}\left(x,y\right)=e^{-j\Phi (u,v)}k^{-2}F_{f}\left[k^{-1}\left(u\;\text{cos}\;\alpha +v\;\text{sin}\;\alpha \right),k^{-1}\left(-u\;\text{sin}\;\alpha +v\;\text{cos}\;\alpha \right)\right]$$ (2)

where Φ(*u*, *v*) is the spectral phase change depending on scaling, rotation and translation. However, the spectral magnitude is translation-invariant,

$$|F_{c}\left(x,y\right)|=k^{-2}\left|F_{f}\left[k^{-1}\left(u\;\text{cos}\;\alpha +v\;\text{sin}\;\alpha \right),k^{-1}\left(-u\;\text{sin}\;\alpha +v\;\text{cos}\;\alpha \right)\right]\right|$$ (3)

The spectral amplitude relationship in Eq (3) indicates that the rotation of the spine results in the same rotation by the same angle in the spectral amplitude images and the scaling by *k* scales the spectral amplitude by *k*^−1^. The rotation and scaling can be further decoupled by defining the spectral amplitudes in the polar coordinates (*θ*, *ρ*). Accordingly, Eq (3) is then expressed as,

$$F_{c}\left(\theta ,\rho \right)=k^{-2}F_{f}\left(\theta -\alpha ,\rho /k\right)$$ (4)

Therefore, the image rotation *α* is then converted as the shift along the angular axis (i.e., horizontal axis in Fig 4E and 4F). The scaling of the original image is converted to a scaling of the radial coordinate (i.e., *ρ*/*k*). By using a logarithmic scale for the radial coordinate, the scaling is then converted to a translation.

$$F_{c}\left(\theta ,\gamma \right)=k^{-2}F_{f}\left(\theta -\alpha ,\gamma -\kappa \right)$$ (5)

where, *γ* = log(*ρ*) and *κ* = log(*k*). Using the polar-logarithmic representation, both rotation and translation are converted to the translations, as shown in Fig 4E and 4F. By applying Fourier transform on the polar-logarithmic representations in Eq (5), one obtains,

$$F_{c}\left(\chi ,\psi \right)=k^{-2}e^{-j2\pi (\chi \alpha +\psi \kappa )}F_{f}\left(\chi ,\psi \right)$$ (6)

where rotation and scaling are represented as phase correlations *e*^−*j*2*π*(*χα*+*ψκ*)^. In the ideal case when two identical images are correlated only with the translation, the inverse Fourier transformation of the phase shifts is a Dirac *δ*-function at (*α*, *κ*). In real cases, the rotation and scaling factors are determined by finding the maximum location from the inverse Fourier transformation image (see Fig 4G). With the same scale and rotation, the phase correlation method is used again to determine the translation factor (*t*_*x*_, *t*_*y*_). The combination of the Fourier and polar-logarithmic transformation is also called Fourier-Mellin method.

### Detection of catheter position and 3D visualization

After the two image modalities are registered into the same coordinate system, 3D rendering occurs in three steps: **1**) The catheter was detected from fluoroscopic images that are captured at two different angles; **2**) The third dimensional location (i.e., the depth information) is calculated from the pair of 2D locations with the known rotation angle; and **3**) The catheter’s position and orientation within the heart is rendered in AR.

#### Detection of catheter from fluoroscopic images

In this study, a 12-French catheter was used for demonstration of the transseptal puncture procedure. Since the catheter is relatively big (4 mm in diameter) and has a higher X-ray absorption rate than the soft tissue and bones, it appears darker than the surrounding area in fluoroscopic images. Therefore, a pixel intensity-based method is developed to determine the catheter’s position. The detection of the catheter starts from the bottom edge of the image with the direction initialized along the Y axis as the catheter is often inserted from the inferior vena cava in the case of a transseptal puncture procedure.

**Fig 5:**
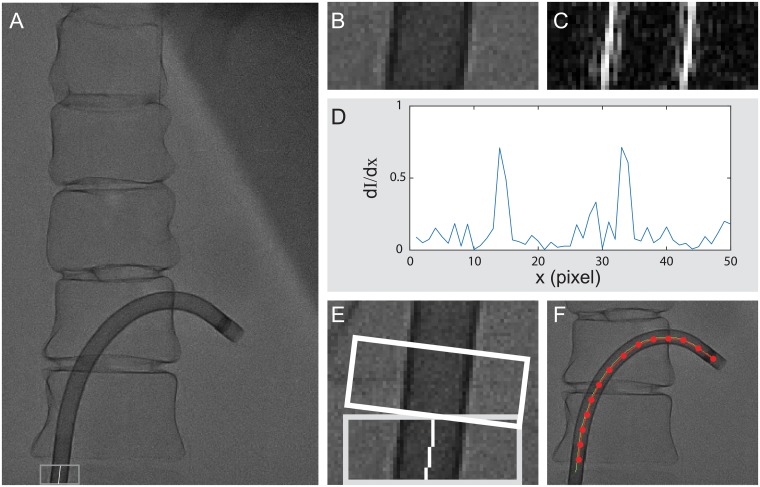
The catheter is detected from the fluoroscopic image. A: Original image. B: The ROI is first extracted at the bottom of the image. C: The gradient of the ROI reveals the edge of the catheter. D: The derivatives of the pixel intensity along the X-axis shows the two edge points are located at the two global peaks. E: An updated ROI is created on top of the previous ROI along the center line of the catheter. F: The final detection results are overlaid to the original image.

The ten rows of pixels at the bottom of the image are first binarized using an adaptive thresholding method. A 50×10 region of interest (ROI) is then extracted with the center located in the middle of the dark object (i.e., the catheter) in the bottom ten rows (Fig 5B). Within the ROI, the edges of the catheter are detected by calculating the derivatives along the direction that is perpendicular to the axial direction of the catheter (Fig 5C & 5D). The centerline of the catheter is then determined as the middle points between two edges. Following the centerline direction, a new ROI is updated on top of the previous ROI and the detection of the catheter centerline is repeated in the updated ROI (Fig 5E). The final results of the detected centerline of the catheter are shown in Fig 5F.

#### Calculation of the 3D position from two fluoroscopic images

**Fig 6:**
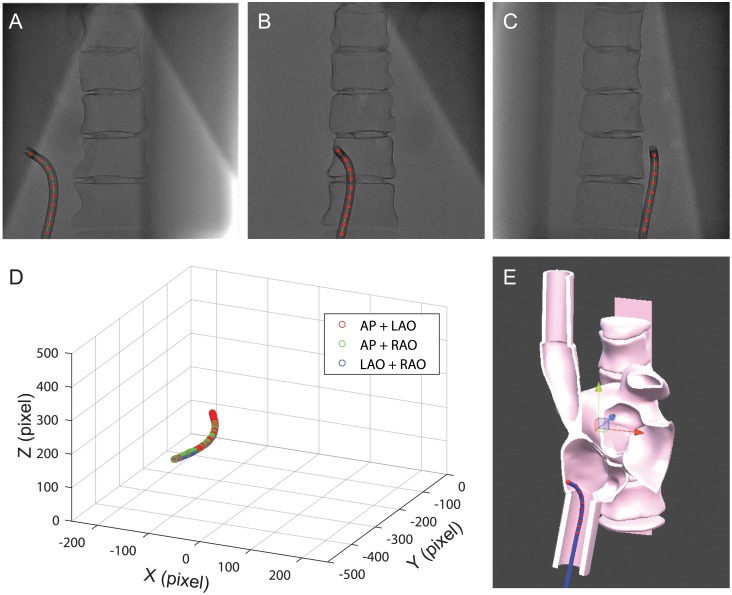
Localization of catheter in 3D space. A, B&C: The catheter is detected from fluoroscopic images that are captured at LAO30, AP, and RAO30 angles. The centerline of the catheter is displayed in red. D&E: The 3D locations of the catheter are determined by using three pairs of images (i.e., AP+LAO, AP+RAO, and LAO+RAO).

The catheter’s position in 3D space is determined by processing the fluoroscopic images captured from two distinct angles. In the experiment, the C-arm was rotated in the axial plane (i.e., around the main axis of the body). Fig 6A–6C show three example images captured with the C-arm oriented at Left Anterior Oblique view 30 degree (LAO30), Anterior-Posterior (AP), and RAO30. Since these three orientations are rotated around the Y axis, the positions of the catheter in the two images have the relationship,

$$P_{1}=[\begin{array}{c} x_{1}\\ y_{1}\\ z_{1} \end{array}]=T_{3d}\cdot P_{2}=[\begin{array}{ccc} \;\text{cos}\;\theta & 0 & \;\text{sin}\;\theta \\ 0 & 1 & 0\\ -\;\text{sin}\;\theta & 0 & \;\text{cos}\;\theta \end{array}][\begin{array}{c} x_{2}\\ y_{2}\\ z_{2} \end{array}]$$ (7)

where *P*_1_ and *P*_2_ are the catheter’s position in the two images, and *θ* is the rotation angle. With the detected position of the catheter (*x*_1_, *y*_1_ and *x*_2_, *y*_2_) and the known rotation angle, the third dimensional positions (**z**_1_, **z**_2_) are solved from Eq (7). Fig 6D and 6E shows the 3D positions of the catheter that are determined from the fluoroscopic images (Fig 6A–6C).

#### Enhanced visualization on augmented reality device

**Fig 7:**
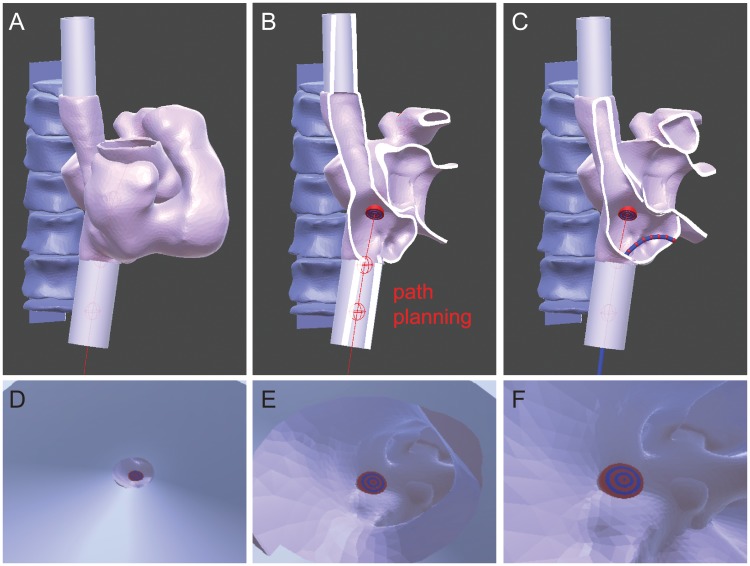
The enhanced visualization on the augmented reality device. A: The 3D rendering of the heart and spine are displayed as holograms on the HoloLens. B: The preprocedural planning is shown as red lines crossing the ideal transseptal puncture site (shown as target rings). C: The catheter is rendered in the 3D space and its position is determined by processing the fluoroscopic images. D-F: A virtual camera is attached to the endpoint of the catheter to provide the first-person view of the catheter when inserting through the inferior vena cava (D), entering the right atrium (E), and approaching the transseptal puncture target (F).

To provide enhanced visualization, the patient’s heart, spine, and catheter are rendered as holograms and displayed on the augmented reality device (Fig 7). In the proposed system, we select the HoloLens (Microsoft Corporation) as the AR device for three reasons: (i) The HoloLens is a pair of video see-through goggles that do not block the surgical view of the medical practitioners and therefore do not disturb the normal interventional procedures. (ii) The HoloLens is able to simultaneously project the results onto the wearable goggles to generate the true stereo holograms for 3D AR visualization. (iii) The HoloLens is integrated with a wireless communication module that can be used to seamlessly receive the processed results from the server computer via a shared Wifi network.

In the first step, the 3D model of the heart and spine are imported into the HoloLens system. The movement trajectory of the catheter was planned by experienced interventionalists and plotted as a virtual path in the 3D model (red lines in Fig 7A & 7B). The target transseptal puncture site was modified as concentric rings to provide additional references.

In addition to the rendering of heart and spine, the catheter position (detected off-line) from nine sets of fluoroscopic images are rendered on the HoloLens. In order to save on computational time, only ten key points are selected and rendered as spherical objects with a diameter of 4 mm (shown as red spheres in Fig 7C). A blue virtual catheter is rendered along the trajectory of the red spherical objects. To assist the operator for a better understanding of the catheter’s location relative to the heart, a virtual camera is also generated at the distal tip of the catheter, with the view angle aligned with the orientation of the catheter’s tip (Fig 7D, 7E & 7F).

The system was also embedded with voice recognition and hand gesture detection. The system allows the operator to use two fingers to grab the 3D renderings and reposition or rotate them to any desired locations or orientations. The 3D renderings can be changed by voice command. When the operator says ‘open’, the system creates a cross sectional view to provide more detailed information about the inner geometry of the heart anatomy (Fig 7B). In addition, several standard views/orientations, such as AP, LAO30 and RAO30, are preloaded in the system and can be easily accessed by voice command (see supplementary video S1 File).

## Results and discussion

The 3D anatomical model of the heart and spine was segmented from the de-identified images from a patient, provided by the Dalio Institute of Cardiovascular Imaging. Using the segmented anatomical models, a phantom model was created by 3D printing (Connex3 Object260, Stratasys Ltd. MN, US) and imaged under X-ray fluoroscopy (Fig 3E and 3F). A thin layer of metal coating was applied to the spine to simulate the contrast of the bone in fluoroscopic images. The use of all medical images was approved by the institutional review board (IRB) at Weill Cornell Medicine.

A catheter (Destino Reach 12 Fr, Oscor Inc. FL, US) was inserted into the 3D-printed model to simulate the transseptal puncture procedure. The experimental setup with varied catheter positions was imaged at 15 fps using an interventional X-ray system (Allura Clarity, Philips Healthcare, US) at the cardiac catheterization laboratories in New York Presbyterian Hospital—Weill Cornell Medicine campus. After image registration and detection of the catheter from the fluoroscopic images, the catheter’s position relative to the phantom heart model was displayed on the HoloLens to provide an augmented visualization.

### Performance of Fourier-based registration

#### Average registration errors

**Fig 8:**
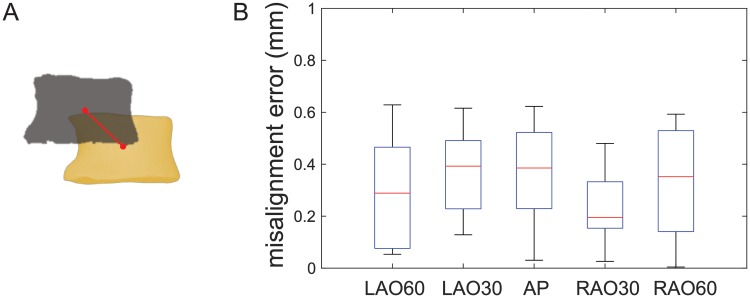
Performance of image registration. A: The misalignment error is defined as the distance between the centroid of the paired vertebrae detected from the fluoroscopic image (in gray color) and projectional CT image (in yellow color). B: The results show that the misalignment errors are consistently below 1 mm for the five experimental groups when capturing the fluoroscopic images at different angles.

The image registration algorithm is evaluated by quantifying the registration errors between the fluoroscopy images and the projectional images derived from the CT model. The registration error is defined as the distance between the centroid of the same vertebrae in the two registered image modalities (Fig 8A). The registration method was tested in five different groups depending on the imaging angles that are anterior-posterior (AP), left anterior oblique view 30 and 60 degree (LAO30, LAO60), and right anterior oblique view 30 and 60 degree (RAO30, RAO60). The experimental results summarized in Fig 8B indicate that the misalignment errors are consistently below one millimeter for all five groups.

#### Comparison with other registration methods

The Fourier based registration algorithm was also evaluated by comparing with other feature-based registration methods. As presented in previous research, the SIFT (scale-invariant feature transform) method was proven effective for registration of medical images of the same modality. Similarly, the SURF (speed up robust feature) method was also applied to extract local features for image registration. Since the SURF method uses an integer approximation of the determinant of the Hessian detector, its computational time is significantly reduced compared to the SIFT descriptor.

In this study, the same fluoroscopic images and CT images were processed for matching local features using the SIFT and SURF methods. The paired feature points were then used to determine an optimal transform matrix. The performance of the two feature-based methods and the present Fourier-based method were first evaluated by measuring the registration success rate. After the two images were registered with the optimal transform matrix, the overlaid images were carefully examined by skilled operators. If there was a significant misalignment (e.g., Fig 8A), the registration was considered a failed case. In total, 35 pairs of fluoroscopic images and projectional CT images were registered by SIFT, SURF, and Fourier based methods. The registration success rate is summarized in Table 1. The results show that the Fourier based method has the highest success rate (100%). While both feature-based methods reveal a lower success rate than the Fourier method, the SURF method performed slightly better than SIFT, which confirms the improved robustness of the SURF detectors and descriptors [20].

**Table 1:** Comparison of registration performance.

registration methods	overall success rate	average registration error (mm)	computational time (s)
SIFT	85.7%	0.883±0.018	1.447
SURF	91.4%	0.875±0.023	1.413
Fourier	100%	0.425±0.021	1.228

The registration precision was also evaluated by measuring the average registration errors for all three methods. The summarized results in Table 1 indicate that the Fourier method has the lowest misalignment error (0.425 vs. 0.883 for SIFT and 0.875 for SURF). The computational time was also measured for the three methods by using a computer running Microsoft Windows Operation System with CPU at 3.10 GHz. The results in Table 1 show that the SIFT and SURF methods have similar performance and consume more computational time than the Fourier based method.

### Detection of catheter and 3D rendering

**Fig 9:**
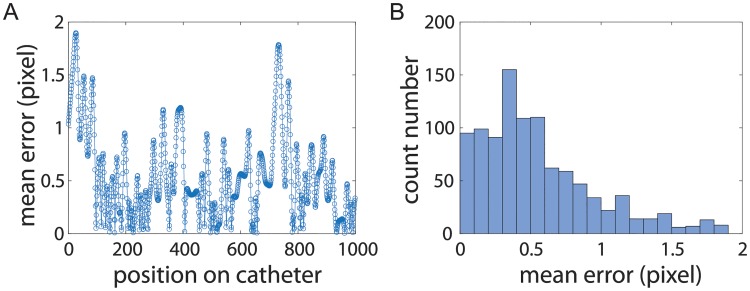
The evaluation results on the determined 3D position of the catheter. A: The mean standard errors for 1000 points on the catheter are all below 2 pixels (i.e., 0.5 mm). B: A histogram indicates the majority of mean standard errors are below 0.5 pixel.

In the experiment, the catheter was inserted into the phantom heart model at fixed positions and imaged from three different angles (i.e., AP, LAO30, and RAO30), as shown in Fig 9A–9C. Although only two different views are needed, the additional view was captured to evaluate/confirm the precision of the determined 3D positions. The paired images captured from two angles were used to calculate the 3D position of the catheter’s endpoint (Fig 9D). The precision of the localization of the catheter in 3D space was quantified by using a mean standard error (MSE),

$$\text{MSE}=\frac{1}{n}\sum_{i=1}^{n}\left|P_{i}-\bar{P}\right|$$ (8)

where, *P*_*i*_ is the calculated 3D position from each paired images and $\bar{P}$ is the mean value of all 3D positions. The evaluation experiment was conducted by placing the catheter at five different locations.

The experimental results on the catheter at an example location indicate that the MSE for 1000 points on the catheter are all below 2 pixels (i.e., 0.5 mm) (Fig 9A). The histogram plot (Fig 9B) demonstrates that the majority of MSE is below 0.5 pixels (i.e., 0.125 mm). The evaluation results for all five locations in Table 2 show that the calculated 3D position has an average MSE of 0.298 mm. To provide context, during a transseptal puncture procedure for an adult patient, an interventionalist expects the catheter to be placed within a ∼5 mm safe region centered at the ideal puncture site [21, 22]. Although the experimental results indicate a high precision on the static phantom model, the effectiveness of using the AR guidance for real-time procedure requires further investigation by using more realistic patient data that involves cardiac and respiratory motion.

**Table 2:** Mean standard errors of 3D position of the catheter.

Group #	L1	L2	L3	L4	L5	Average
MSE (mm)	0.37	0.39	0.43	0.12	0.18	0.29
STD (mm)	±0.23	±0.27	±0.26	±0.08	±0.13	0.19

### Discussion

In the present AR image-guidance system, the 3D position of the catheter is determined by processing two fluoroscopic images. Using the spine as the fiduciary marker, the image registration method is able to set the 3D heart rendering (segmented from CT images) and the catheter rendering (determined from fluoroscopic images) in the same coordinate system.

The visualization of the patient’s anatomic models has been adopted in clinics for the diagnosis of diseases, planning of surgical procedure, and intraoperative guidance. However, the visualizations are typically displayed on 2D computer screens. In order to obtain an intuitive perception of the 3D models, the operator needs to frequently rotate the models by using a computer mouse. The development of AR and VR technologies enables a more intuitive solution for 3D visualization and provides a transformative human-machine interface. The smart AR glasses used in the present system allow the user to use both eyes to directly view the 3D models instead of looking at a 2D screen while constantly rotating the models. Moreover, the virtual 3D heart models can be rendered at any position/orientation that is comfortable to the interventionalists. Compared to the conventional 2D visualization system that requires the user to frequently switch back and forth between the display monitor and surgical sites, the direct projection/rendering enabled by AR technology allows for an improved hand-eye coordination.

The limitations of the proposed system are the following: **(i**) The 3D printed heart model is static, and therefore does not recapitulate the dynamic geometry and lcoation of the heart due to contractions and respiration. Future work will need to overcome these challenges by gating the CT model with multiple phased CT scans with an ECG signal. Furthermore, other real-time changes in cardiac phase, heart rate, volume status/loading conditions can affect the true cardiac position and geometry. Previous literature has shown that real-time phase matching between 2D fluoroscopic images and 3D CT images is achievable and can compensate for respiratory motion [23]. Similarly, we can generate the pseudo 4D CT images of the heart by analyzing motion vector fields [24]. The pseudo 4D images can facilitate the rendering of a dynamic heart model that well reflects the real-time geometry and position of the heart. Another solution could incorporate ultrasound that can directly image the heart tissue. These future improvements will directly dictate the accuracy of this image-guidance system, which currently is unknown, since only the precision of catheter localization has been characterized for the proposed system. **(ii**)Updates for renderings can only be taken as quickly as a C-arm can switch between the two fluoroscopic angles. This precludes the rendering of continuous motion of the catheter. However, continuous tracking is possible for hospitals that have bi-plane C-arms. **(iii**) Difficulty of automated segmentation of spine exists in processing real patient fluoroscopic images. Currently we tested our proposed system on a 3D-printed model that admittedly has greater contrast of the spine features compared to real patient images that have more complex tissue structures. More advanced image processing and potentially machine learning algorithms will be needed to effectively segment spines in real-time on patient images.

## Conclusion

This paper reports a novel AR-assisted 3D visualization system for image guided percutaneous cardiac interventional procedures. The 3D model of the patient’s heart was segmented from CT images for planning the surgical procedures. A Fourier based method was capable of registering the intraoperative fluoroscopic images with the 3D heart model. Compared to other feature-based methods, the registration algorithm based on the polar-logarithmic transform of the frequency domain showed a significantly increased registration success rate (100% vs. 85.7-91.4%) and improved registration accuracy (0.42 vs. 0.88 mm). The 3D positions of the catheter were calculated by processing the fluoroscopic images captured at different angles. The detected catheter was precisely rendered as holograms on the AR device in 3D space with an MSE of 0.30 mm.

Compared to standard interventional technology, the AR system enables the 3D visualization and more user-friendly interface for interventionalists to better understand the heart anatomy. Therefore, it may assist the development of new therapeutic procedures and lower the learning curve for existing procedures. Furthermore, the developed guidance system and benchtop models can be used for pre-procedural planning and practicing complicated procedures. The new system can also work as a new teaching/training tool for residents and fellows and provides a quantitative platform for comparing methods for different PCI procedures amongst interventionalists.

## Supporting information

S1 File AR guidance system for percutaneous cardiac intervention. The supplementary video demonstrates the use of the AR system to guide the catheter insertion for the transeptal puncturing procedure. The 3D rendering of the heart is dynamically changed according to the user input via gesture or voice command. A virtual display is also created to provide a first-person view to assist the navigation of the catheter inside the heart.
(MP4)

S2 File Data and programs. The compressed file contains an anonymized data set and matlab programs necessary to replicate this study.
(RAR)
